# Implicit Belittlements Call for Implicit Measures: Emotional Reactions to Youth Paternalistic Stereotypes

**DOI:** 10.5334/pb.351

**Published:** 2017-07-07

**Authors:** Aude Silvestre, Johanne Huart, Benoit Dardenne

**Affiliations:** 1 Fonds National de la Recherche Scientifique (F.R.S. – FNRS), University of Liège, BE

**Keywords:** paternalistic stereotypes, affect, emotional Stroop task, social sharing of emotions

## Abstract

Age discrimination at work can potentially affect every worker. Indeed, like ‘old’ workers, young ones hired in their first job elicit the idea that they have quite interesting social abilities but lack of competence, which constitutes a case of paternalistic stereotypes (Fiske, Cuddy, Glick, & Xu, 2002). Generally, the negative (incompetence) facet of such stereotypes is not blatantly expressed, but is subtly conveyed behind an apparently positive discourse. Consequently, it is considered as being generally under-detected, while harmful. In this paper, we examine whether paternalistic stereotyping’s under-detection is real or if it is due to the use of inadequate measures. Based on a study showing that targets *feel* that something is wrong (Dardenne, Dumont, & Bollier 2007), we rely on affective measures to investigate whether the detection of the subtly conveyed negative facet of paternalistic stereotypes calls for subtle, implicit measures. In Study 1, explicit self-reports of targets’ affective states after a meeting with a paternalistic boss revealed mainly positive affect. In Study 2, an implicit emotional measure however revealed the presence of a negative affective state. The last Study, using a more ecological affective measure, demonstrates that paternalistic stereotypes trigger an ambivalent affective reaction. Altogether, the three studies suggest that the negative facet of paternalistic stereotypes is not as under-detected as we thought.

## Introduction

Overt negative stereotyping of individuals is often socially inappropriate, even legally punishable. Unfortunately, even though instances of blatant stereotyping have become less common, the act of judging individuals negatively because they belong to a certain social group is still just as topical as ever (Clark & Gochett, 2006; D’Augelli & Hershberger, 1993; Klonoff & Landrine, 1995; Swim, Cohen, Hyers, Fitzgerald, & Bylsma, 1997). For instance, Swim, Hyers, Cohen, and Fergusson (2001) found that women reported experiencing or witnessing one or two sexist episodes per week. In another survey study, only 11% of the participants reported that they never heard derogatory remarks about African Americans, while 52% reported hearing those remarks often or frequently (Swim et al., 1997). The permanence of stereotyping and discrimination despite their social reproof has been rendered possible by a change in the form in which they are expressed (which is paradoxically a consequence of social reproof). Nowadays, they are more subtle and less obvious. Research has proved this is the case for racism, for instance, under the form of what is called modern racism (McConahay, 1986) or aversive racism (Gaertner & Dovidio, 1986). It is also the case for sexism, under the disguise of modern sexism (Swim, Aikin, Hall, & Hunter, 1995) or paternalistic stereotypes (Fiske et al., 2002; Glick & Fiske, 1996).

At work, a prevailing discrimination factor is age. In Europe, 58% of workers perceive age discrimination as a widespread problem in their country (European Commission, 2009, cited in Krings, Sczesny, & Kluge, 2011). Actually, surveyed workers reported experiencing age discrimination more often than other forms of discriminations (Snape & Redman, 2003). In Belgium, age discrimination in the workplace or during job interviews is that spread that it has attracted the attention of the *Centre for Equal Opportunities and Opposition to Racism*, which ordered an opinion poll (Spaas & Vandenbroucke, 2012). It indicates that at least 8% of the respondents, during the last 5 years, felt discriminated against because of their age.

While research about the widespread age discrimination in the professional field mainly concerns ‘old’ workers (Brooke & Taylor, 2005; Desmette & Gaillard, 2008; Kite, Stockdale, Whitley, & Johnson, 2005; Krings et al., 2011; Rupp, Vodanovich, & Crede, 2006, see also Loretto, Duncan, & White, 2000; North & Fiske, 2012), an international survey reported by Krings et al. (2011), indicates that *young* workers also feel discriminated against based on their age (see also Snape & Redman, 2003). Indeed, although discrimination is experienced similarly amongst young and older workers, it is mainly the 18 to 34 years-old category that reported facing age discrimination during the hiring process, decisions in promotions attribution or in lay-off (sees also Snape & Redman, 2003). Similarly, Loretto et al. (2000) found that 35% of their student respondents reported having experienced age discrimination while working part-time or during the vacations. Given the prevalence of age discrimination at work and the scarce research tackling the nonetheless present discrimination against young workers in the workplace, research about young workers’ stereotypes is needed. Following the example of sexism and racism, we suggest that age discrimination at work is not only expressed blatantly, but that stereotypical beliefs about young workers can be conveyed using more subtle ways.

### Paternalism

According to the Stereotype Content Model (SCM) all stereotypes can be classified alongside 2 orthogonal dimensions (forming 4 quadrants or sub-stereotypes): the warmth dimension and the competence dimension. Paternalistic stereotypes appear when social groups and their members are seen as very nice and sociable – high on the warmth dimension – but quite incompetent – low on the competence dimension (Fiske et al., 2002). Research has repeatedly shown that stereotypes about the elderly fall into this category, while younger people (adults) are seen as more competent (Cuddy, Fiske & Glick, 2008; Fiske et al., 2002). Nevertheless, it is important to note that most of the research focused on older/younger adults in general, not on workers in particular. If it has been shown that older employees are believed to be less effective than younger employees in various job-related tasks (Avolio & Barrett, 1987; Rosen & Jerdee, 1976a, b; Singer, 1986), nothing is said of a similar comparison with, for instance, young and inexperienced workers recently recruited for their first job. The necessity of subtyping wide social categories has already been outlined. For example, Walzer and Czopp (2011) recently pointed out the necessity of subtyping groups according to the various acceptations of competence (e.g., intelligence, talent). Cuddy and Fiske (2002) themselves mentioned the necessity of subtyping age categories. The only study we found comparing old and young *workers* on warmth and competence dimensions (Krings et al., 2011) revealed that, if the first are evaluated as less competent and warmer than the latter, differences are tenuous and both groups would fall into the same quadrant of the model if they were located on the bi-dimensional graph. Daily examples seem to confirm this idea that (very) young and inexperienced workers are, as are older ones but for different reasons, the target of paternalistic stereotypes.

A first example can be found in the (probably well-intended) British Safety Council “Speak Up, Stay Safe” campaign,^1^ which identified qualities that makes young workers vulnerable from work related accidents. Sentences such as: “In many cases lack of information, lack of work experience and lack of confidence are to blame” or “Some workers may have particular requirements, for example new and young workers, new or expectant mothers, and people with disabilities” can be found on the campaign website. While not openly mentioned, the incompetence of young professionals shows through the notion of inexperience or the association with disabled people. The precautions are at first sight gentle, aiming to help young people and to prevent them from injuries, but implicitly, they convey the message that they are not competent. The notion of incompetence also shows through Belgians’ stereotypes about young professionals, described as ‘unmotivated, undisciplined, exacting, unreliable, inexperienced, overconfident, but having good communication abilities, a dynamic disposition and a willingness to learn’ (Spaas & Vandenbroucke, 2012). In a very large New Zealand survey, both older union members and employers were asked to think about workers in different age groups and to indicate which groups best illustrated a number of qualities and factors (McGregor & Gray, 2002). The authors observed that while young workers were evaluated quite high on enthusiasm, they were at the same time evaluated as lacking judgment, innovation, credibility, professionalism, and people skills, amongst other qualities.

Whereas, as aforementioned, research on paternalistic stereotyping experienced by young professionals has been very scarce, paternalism has nonetheless attracted the attention of researchers, particularly in the domain of sexism. Studies have demonstrated that, despite its apparent positivity, the notion of incompetence subtly conveyed by paternalistic sexism has negative effects on its targets (e.g., Becker & Wright, 2011; Dardenne et al., 2007; Dumont, Sarlet, & Dardenne, 2010). However, despite its deleterious consequences, women generally accept it (Moya, Glick, Expósito, de Lemus, & Hart, 2007). This acceptance has been told to be partly due to the fact that paternalism’s positive tone makes its negative side hard to detect (Barreto & Ellemers, 2013; Swim, Mallett, Russo-Devosa, & Stangor, 2005). However, difficulties to clearly identify paternalistic sexism as such do not mean that women do not notice that something is wrong when confronted with it. Indeed, women have been shown to feel ill at ease in paternalistic work-related situations, which they reported to be less pleasant than control ones and as equally unpleasant as hostile sexist situations (Dardenne et al., 2007, Experiment 2), while at the same time they do not report explicitly sexist discrimination. Consequently, targets’ acceptance of paternalism may not indicate that they do not detect it.

In this paper, we propose that the targets of paternalistic stereotypes are able to identify them in their negative form but do not or cannot systematically report it. Hesitations to explicitly reporting prejudice has been evidenced to be linked to fear of social cost. Research has shown that individuals who attribute a negative experience to prejudice are perceived as overreacting (Czopp & Monteith, 2003), as complainers (Kaiser & Miller, 2001) or troublemakers (Kaiser & Miller, 2003). We propose an additional explanation. More precisely, we suggest that the positive side of paternalistic stereotyping related to warmth would be easily detected, positively experienced, and the resulting affective reactions would be reported without difficulty using traditional affective self-report measure. However, the negative side of paternalism, related to incompetence, far from being undetected, would be negatively experienced but would need subtler means to be evidenced. Studies have shown that attitudes, stereotypes, or emotional states are not systematically well identified using only self-reports and that self-reports are not systematically related to less blatant, covert or implicit measures (Carney, Banaji, & Krieger, 2010; Meagher, & Aidman, 2004; Quirin, Kazén, Rohrmann, & Kuhl, 2009). For instance, Bosson, Haymovitz, and Pinel (2004) showed that although participants did not report feeling anxious in a stereotype threat situation using a self-report method, a non-verbal measure of anxiety did indicate that participants demonstrated anxiety in that particular situation.

## Our Studies

In this paper, building on Dardenne et al. (2007)’s study revealing women’s uneasiness in paternalistic work-related situations, we investigate affective reactions to paternalistic stereotypes. Contrary to the idea that paternalism acceptance could be due to the fact that paternalism’s positive tone makes its negative side hard to detect (Barreto & Ellemers, 2013), we propose that the negative facet is indeed detectable but not blatantly reported, hence calling for more implicit measures. Considering the stereotype of (very) young and inexperienced workers as a case of – particularly understudied – paternalistic stereotypes, we chose to rely on this stereotype across the following 3 studies. Therefore, the aim of this paper is to investigate more implicit ways to pinpoint affective reactions of (very) young professionals exposed to paternalistic stereotyping in a work-related context. In three studies, we use the targets’ affect to investigate their potential detection of the negative side of paternalistic stereotypes, and the conditions in which targets express emotional reactions.

First, based on studies that report no explicit negative reactions to paternalistic sexism (Barreto & Ellemers, 2005; Dardenne et al., 2007), we posit that participants will fail to report any affective reaction related to the negative, more subtle, side of paternalistic stereotyping using solely self-report measures. In Study 1, we exposed young participants to a work-related situation tainted with youth paternalism, then measured their positive and negative affective reactions using Likert-type scales. We expected participants to report more positive than negative emotions when confronted with paternalism, as opposed to a hostile condition.

Second, based on the fact that the negative side of paternalism is expressed implicitly, we suggest that using an implicit emotional measure would confirm that the experience of paternalistic stereotyping is not lived as positively as it could seem at first. In Study 2, we used an emotional Stroop task – which is largely used to study attentional bias towards affective self-relevant words and indicating the current affective state – to investigate whether using an implicit measure would reveal some negative affects after a paternalistic episode. More precisely, we predict an attentional bias towards negative words for participants confronted with paternalistic instructions (that is, longer reaction times for these participants than for those confronted with neutral instructions).

In Study 3, we propose a more ecological means to identify affective reactions to paternalistic stereotyping in a work-related context. Literature on Social Sharing of Emotion (SSE) reports large evidence that any emotional episode, even of mild intensity, leads people to share it and talk about it (Luminet, Bouts, Delie, Manstead, & Rimé, 2000; Rimé, 2009a, 2009b). We use this natural need people have to share emotions with close others as a more subtle (compared to Study 1) as well as more ecologically valid (compared to Study 2) measure of affect triggered by an episode of paternalistic stereotyping. Studies using SSE mainly focus on the diffusion of the emotional event (how many people the event was shared with, how often it was shared, etc.). However, analysing the content of SSE after an episode of paternalistic stereotyping offers another way of understanding the affective reactions of participants confronted with youth paternalism. We expected participants confronted with paternalism to report as many negative affective reactions compared to a hostile condition, reflecting that something “not that good” was going on under paternalistic instructions. Alternatively, since cognitive clarification (finding explanations) is a function of SSE (Rimé, 2007) and that paternalism is ambivalent by nature, we could also expect participants to socially share equivalent amounts of negative and positive emotions. Indeed, confrontation with both a positive message of warmth and a negative message of incompetence simultaneously might lead participant to feel somewhat ambivalent.

### Ethics statement

All the studies were carried out in accordance with the Declaration of Helsinki. The studies received ethical approval from the Ethics Committee of the Department of Psychology and all participants provided written informed consent after the nature of the procedures had been fully explained.

## Study 1

### Methods

#### Overview

Participants were told that they were about to participate in a study examining the reactions people can have on the first day of their first job. They started by completing an informed consent form and demographic questions. They were then presented with a scenario describing their hypothetical meeting with their new boss and colleagues on their first day. The scenario was either a paternalistic, hostile, or neutral description of the situation. Finally, explicit self-reports of positive and negative emotions were collected. Participants were then thanked and fully debriefed.

#### Participants

Participants were 68 (36 female) under- and post-graduates (mean age = 20.90; *SD* = 3.07) who were native French speakers. They were approached in different places on campus. If they agreed to participate, the experimenters gave them one of the three questionnaire packages at random (*N* = 24 for paternalistic, *N* = 22 for hostile, and *N* = 22 for neutral scenarios). Power analysis assuming medium effect size (f = .25) at alpha = .05 indicated that *n* of 68 would provide around 80% power (G*Power 3.1.9.2; Faul, Erdfelder, Buchner, & Lang, 2009).

#### Hypotheses

Hyp. 1a: participants’ reports in the paternalistic and control conditions will reveal more positive emotions than participants in the hostile condition.

Hyp. 1b: participants’ reports in the hostile condition will reveal more negative emotions than participants in the paternalistic and control conditions.

Hyp. 1c: participants’ reports in the paternalistic condition will reveal significantly more positive than negative emotions.

Hyp. 1d: participants’ reports in the hostile condition will reveal significantly more negative than positive emotions.

#### Procedure and measures

In the scenario, participants read a description of their alleged first meeting with their new boss and colleagues. They were asked to imagine that they were welcomed by the director of the company. The text started with the same sentences across conditions: “Imagine that you have been hired by a company (*Global*) soon after graduation. You have arrived on your first day and the director, Mr. Delloy, receives you in his office for his welcome speech. Here is a description of your meeting: Upon my arrival at the director’s office, his secretary let me in. The director invites me to sit down, what I do. After offering me something to drink, he starts to talk: “First of all, let me wish you welcome into *Global*”. The following text constituted a paternalistic, hostile, or neutral description of the company. In the paternalistic version, the boss was protective, benevolent, helpful and somehow intrusive (in his employees’ professional and personal lives). The boss used sentences like: “Our Company is like a big family. I’m the father – authoritarian but protective – and the employees are like my children – obedient and grateful. In our company, we are aware that we need to hire young workers because they are our future, much as children are adults’ future. It is true that young workers are quite inexperienced but our older and more experienced colleagues are there to support and help them and take charge if needed. Here, at *Global*, we are used to caring for each other, especially the youngest ones, because everyone’s happiness is essential for our company to work well.” In the hostile version, the boss openly expressed all his negative stereotypes about young workers (inexperienced, reckless, lazy, greedy, etc.; see Chasteen, Schwarz, & Park, 2002). Hostility towards young workers was expressed in sentences such as “The employees are not here to babysit the youngsters. Avoid wasting their time with stupid questions that you, young people often ask” or “Young people are all the same, hypocrites and profiteers.” In the neutral version (control condition), the director described the company structure (departments, personnel, products, etc.) in neutral words. At the end of the description, participants read that they were meeting their colleagues. They were described as being paternalistic, hostile, or neutral towards the participants, using sentences similar to the ones used by the boss. At the end of their reading, they were asked to take a few moments to think about the meeting and their feelings.

Participants were then asked to rate to what extent they were currently experiencing 16 positive (e.g., confident, optimistic, happy) and 34 negative^2^ (e.g., worried, angry, sad) emotions, using a 7-point Likert scale, from 1 (*not at all*) to 7 (*totally*). We ran a factor analysis on all of the emotional items. Two factors were extracted, which explained 53% of the total variance. The emotional items with loadings higher than .45 were kept and we created one positive (8 items; e.g.; optimistic, enthusiastic, calm) and one negative score (13 items; e.g., angry, worried, frustrated). The emotional scores presented a good internal consistency, α = 91 for positive emotions and α = .94 for negative emotions. Participants then completed a manipulation check assessing the paternalistic and/or hostile tone of the text describing their meeting with the company’s director and colleagues. They were asked how paternalistic, protective, hostile and aggressive they found the meeting, the director and the colleagues, separately, using a 7-point Likert scale, ranging from 1 (*not at all*) to 7 (*totally*). We created an index of paternalism (α = .90) and hostility (α = .96) by computing the means of the six corresponding items (paternal and protective or hostile and aggressive – for the meeting, the director and the colleagues).

### Results and Discussion

Analyses revealed no significant effect due to participants’ gender, and therefore the reported results do not include gender as a factor.

#### Manipulation check

The paternalistic condition was perceived as more paternalistic (*M* = 5.31, *SD* = 1.34) than the control (*M* = 3.77, *SD* = 1.19), and hostile conditions (*M* = 1.88, *SD* = 1.04), with both *t*s > 4.10, *p*s < .001, and Cohen’s *d*s > 1.21. The last two conditions differed significantly from each other, *t* (42) = –5.61, *p* < .001, *d* = 1.69, with the control condition evaluated as more paternalistic than the hostile condition. Similarly, the hostile condition (*M* = 5.63, *SD* = 1.29) was perceived as more hostile than the paternalistic (*M* = 1.39, *SD* = .58), and control conditions (*M* = 1.64, *SD* = .74), with both *t*s > 12.61, *p*s < .001, and Cohen’s *d*s > 3.79. The latter two conditions did not differ significantly from each other, *t* (44) = –1.28, *p* = .21; *d* = –.36.

#### Emotional self-reports

A 3 (condition: Paternalistic vs. Hostile vs. Control) × 2 (emotion valence: Positive vs. Negative) ANOVA, with valence as within-subject variable, revealed a main effect of valence, *F* (1, 65) = 19.86, *p* = 001, η^2^_p_ = .23. Participants reported more positive (*M* = 3.57, *SD* = 1.39) than negative emotions (*M* = 2.62, *SD* = 1.45), *t* (67) = 2.98, *p* < .01, Cohen’s *d* = .67, 95% CI [.32, 1.60]. More importantly, the predicted interaction between valence and condition was significant, *F* (2, 65) = 50.58, *p* < .001, η^2^_p_ = .61.

**Figure 1:**
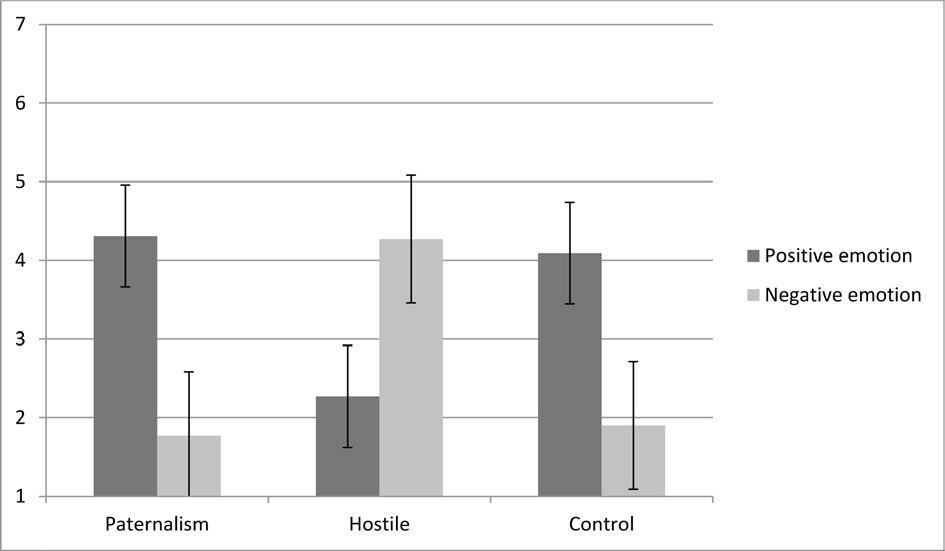
Means for positive and negative emotions by experimental condition (Study 1).

To better understand this interaction (see Figure 1), we performed a one-way ANOVA on positive and negative emotions separately with condition as between-subject factor. Confirming Hyp. 1a, results revealed, for positive emotions, a main effect of condition, *F* (2, 65) = 28.25, *p* < .001, η^2^_p_ = .44. Post hoc analyses using the Bonferroni test indicated that participants in the paternalistic (*M* = 4.31, *SD* = .94) and control conditions (*M* = 4.09, *SD* = 1.35) reported significantly more positive emotions than those in the hostile (*M* = 2.27, *SD* = .84) condition, with both *p*-values < .001. The mean score for reported positive emotions did not differ significantly between the paternalistic and control conditions, *p* = 1. Regarding negative emotion, the one way ANOVA revealed a main effect of condition as well, *F* (2, 65) = 55.72, *p* < .001, η^2^_p_ = .63. Post hoc analyses using the Bonferroni test indicated that participants in the paternalistic (*M* = 1.77, *SD* = .68) and control conditions (*M* = 1.90, *SD* = 1.01) reported significantly fewer negative emotions than those in the hostile (*M* = 4.27, *SD* = .97) condition, with both *p*-values < .001 (Hyp. 1b). The mean score for reported negative emotions did not differ significantly between the paternalistic and control conditions, *p* = 1.

To compare the level of positive and negative self-reported emotions within each condition, we performed paired t-tests. As predicted, participants in the paternalistic condition reported more positive than negative emotions, *t* (23) = 8.59, *p* < .001, *d* = 3.10, 95% CI [1.39, 3.16]. As in the paternalistic condition, participants in the control condition reported more positive than negative emotions, *t* (21) = 4.87, *p* < .001, *d* = 1.84, 95% CI [1.26, 3.13], (Hyp. 1c). In the hostile condition, participants reported more negative than positive emotions, *t* (21) = –6.62, *p* < .001, *d* = 2.20, 95% CI [–2.63, –1.37], confirming Hyp. 1d.

As predicted, participants reported more positive than negative affects when exposed to paternalistic stereotyping. Participants in a situation where no stereotyping was present presented the same affective pattern than participants in the paternalistic condition, i.e., they reported higher levels of positive, compared to negative, affects. The results confirmed our hypothesis that the sole use of explicit self-reports prevents participants to report any affective reaction related to the subtly implied negative side of youth paternalism. As stated earlier, previous studies have reported increased difficulty for explicit self-reports to identify specific emotional states (Bosson et al., 2004).

Nevertheless, we think the participants may have somewhat sensed that something was wrong in the paternalistic situation because their emotional pattern was similar to the neutral situation, where no kindness was expressed. One might think that positive emotions could have been higher following a paternalistic encounter than a neutral encounter, given that paternalism is supposed to feel good. But this was not the case. The experience of paternalism in our study took place in a professional context and it has previously been shown that paternalistic comments in a work context triggered a small level of positive reactions towards the paternalistic perpetrator (Moya et al., 2007). We suggest that this may be a clue that paternalism is not all about kindness and care after all. Anterior research has also evidenced that attitudes or concepts measured via explicit instruments are rarely associated to those measured using implicit instruments (Carney et al., 2010; Fazio, Jackson, Dunton, & Williams, 1995; Quirin et al., 2009). In our second study, we aimed to show that the use of an implicit task will reveal a different affective pattern than the one found using explicit self-reports. We expected that following a paternalistic encounter, participants will present attentional bias towards negative words, which would reveal, according to the Stroop literature (Gotlib & McCann, 1984; Watts, McKenna, Sharrock, & Trezise, 1986; Williams & Nulty, 1986), that negative words were more accessible to the participants confronted with paternalism, suggesting that participants are far from being incapable of perceiving the subtle negative tone of paternalism. No such bias is expected in a situation were no stereotypes are expressed. We expect that when using a more appropriate measure, participants’ affective reactions can be evaluated.

## Study 2

### Methods

#### Overview

Participants were told that they were about to participate in a study examining the reactions people can have to their first job interview. They started by completing an informed consent form and demographic questions. They were then presented with a scenario describing a hypothetical job interview. The scenario was either a paternalistic or a neutral description of the situation. Participants then completed an emotional Stroop task before being thanked and fully debriefed.

#### Participants

Participants were 40 (20 female) under- and postgraduate students who were native French speakers (mean age = 22.05, *SD* = 2.35). They were approached in different places on campus. If they agreed to participate, they were invited to follow the female experimenter to the laboratory. Participants were then randomly assigned to a paternalistic or control condition (*N* = 21 and 19, respectively). Power analysis assuming medium effect size (*d* = .50) at alpha = .05 (one tail) indicated that *n* of 40 would provide around 57% power (G*Power 3.1.9.2; Faul et al., 2009).

#### Hypothesis

Hyp. 2: Participants in the paternalistic condition will present an attentional bias towards negative words, while participants in the control condition will not. More precisely, the interference index for negative words will be higher amongst participants confronted with a paternalistic speech, compared to participants confronted with a neutral speech.

#### Procedure and measures

***Paternalism induction.*** Participants were asked to imagine they were searching for a job after graduation. After several months of searching, following their application for a job in a company (*Global*), they were invited to meet the person in charge of recruitment for the company. A description of the hypothetical meeting was written down. The person in charge of recruitment either described the company as being paternalistic towards young workers or explained the company’s structure in neutral terms. The scenario started with the same sentences in both conditions: “Imagine that you have been looking for a job for several months now. You recently applied for a job and you have been invited for a job interview. You are waiting for your interview when the person in charge of the recruitment invites you in. Here is a description of your meeting.” What followed was the paternalistic or neutral description of the company. In the paternalistic version, the recruiter presented the director and colleagues as protective, benevolent, helpful, and somehow intrusive. The recruiter used sentences similar to those in Study 1. In the neutral version (control condition), the recruiter described the company’s structure (departments, personnel, products, etc.) in neutral words. At the end of the description, participants read that they were about to take the selection test. Before taking the test, they were asked to take a few moments to think about the meeting and their feelings.

***Emotional Stroop task.*** The Emotional Stroop task has been developed to examine cognitive processing associated with emotional disturbance, and used to measure construct accessibility (Gotlib & McCann, 1984; Williams et al., 1986), for instance. The Stroop effect is present when the color-naming latency is slower for emotional words compared to neutral words. The Emotional Stroop task has been largely used to study individuals with high level of anxiety (Mathews & McLeod, 1985; Richards, French, Johnson, Naparstek, & Williams, 1992) but also with depression (Gotlib & Cane, 1987), and phobias (Watts et al., 1986).

The task consisted in the random presentation of 5 positive emotional words (e.g., *sympathy, competence*), 15 negative emotional words (e.g., *unease, perplexity*) and 10 neutral words (e.g., *bottle, curtains*) of similar length. Each word was presented on a white background, with the key-color combination appearing at the top of the screen. Participants had to press on the keyboard the corresponding key (*d* for red words, *f* for green words, *j* for blue words, and *k* for black words). The data were recorded using Inquisit by Millisecond Software (v. 3.0.2.0., 2008). Each word was presented once in each color (black, green, blue and red). This produced a total of 120 trials. The color in which each word was presented was randomly determined. The task was to correctly identify the color of the word. The time each participant took to answer was recorded in milliseconds; the number of correct and incorrect answers was also recorded. To deal with outliers, median reaction times were used. Based on the Emotional Stroop literature (Mathews et MacLeod, 1985; Richards et al., 1992; Williams, Mathews, & MacLeod, 1996), to examine an interference effect on negative (positive) words, we created a reaction time difference index by subtracting the median reaction time to identify the colors of neutral words from the median reaction time to identify the colors of negative (positive) words. A positive score means that it takes more time to identify the color of a negative (positive) word than of a neutral word. Conversely, a negative score means that it takes more time to identify the color of a neutral word than of a negative (positive) word. Finally, a null score means that it takes the same time to identify the colors of negative (positive) and neutral words.

Participants then completed a manipulation check assessing the tone of the text describing their meeting with the recruiter. They were asked to say how paternalistic or neutral they found the meeting and the recruiter, separately, using a 7-point Likert scale, ranging from 1 (*not at all*) to 7 (*totally*). We created an index of paternalism (*r* = .83) and neutrality (*r* = .93) by computing the means of the corresponding items.

### Results and Discussion

#### Manipulation check

As expected, the paternalistic condition was evaluated as more paternalistic (*M* = 4.62, *SD* = 1.91) and less neutral (*M* = 2.67, *SD* = 1.73) than the control condition (*M* = 2.74, *SD* = 1.59, *t* (38) = –4.49, *p* < .001; Cohen’s *d* = 1.42; and, *M* = 5.16, *SD* = 1.77; *t* (38) = 3.36, *p* = .002; Cohen’s *d* = 1.07).

#### Emotional Stroop

To test our hypothesis that there would be an interference effect towards negative words in the paternalistic condition, compared to the control condition, we performed two t-tests. The results showed that the interference index for negative words is higher in the paternalistic condition (*M* = 32.93, *SD* = 80.11) than in the control condition (*M* = –22.79, *SD* = 82.82), *t* (38) = 2.16, *p* = .037; Cohen’s *d* = .68, 95% CI [3.54, 107.89], therefore confirming Hyp. 2. As for the interference index for positive words, the paternalistic condition (*M* = 14.48, *SD* = 87.04) did not differ significantly from the control condition (*M* = 1.42, *SD* = 131.24), *t* (38) = .37, *p* = .71; Cohen’s *d* = .12, 95% CI [–57.58, 83.70]). To check whether such non-significant results were due to a lack of statistical power, we conducted power analyses with power set at 75% and α = 05. Sample sizes would have to increase up to 1930 in order for group difference to reach statistical significance. Thus, it is unlikely that our negative findings can be attributed to a limited sample size. Study 2 revealed that participants faced with a paternalistic situation took more time to identify the color of a word when the word was negative than when it was neutral, unlike participants in a control condition. No such bias appeared for positive words. As predicted, the use of implicit emotional measures confirmed that paternalism is negatively experienced. Study 2 provided evidence that not expressing discomfort after an episode of paternalism does not reflect the individual’s failure to detect it but can be attributed to the use of inappropriate measures to capture it. In a desire to replicate these findings using a more ecological measure of emotions, we decided to use the natural proclivity of people to share their emotions following an emotional episode. In the third study, we used SSE as a way to examine emotional reactions and expressions after an episode of paternalistic stereotyping, not by looking at the diffusion of the emotional event but by analyzing the content of the sharing. As in Study 1, we expected that participants would report only the obvious positive side of paternalism on self-reports measures. However, they would express the negative side of paternalism using the SSE measure. More specifically, whereas the pattern of emotional reaction in a hostile and a control condition would be the same in the self-reports as in social sharing, the pattern of emotional reaction in the paternalistic condition would differ: participants are expected to report more positive than negative emotions in their self-reports but more negative than positive emotions in their social sharing, therefore revealing the negative side of paternalism. As introduced earlier, the ambivalent nature of paternalism also encourages us to propose the sharing of a somewhat ambivalent reaction, which would translate into similar reports of negative and positive emotions.

## Study 3

### Methods

#### Overview

The procedure and the scenarios used in Study 3 are very similar to the ones used in Study 1, but a measure of emotional social sharing was added.

#### Participants

Participants were 132 (66 female) under- and postgraduate students (mean age = 21.53; *SD* = 2.06) who were native French speakers. Participants were randomly assigned to a paternalistic, hostile or neutral context (control condition) (*N* = 45, *N* = 43, *N* = 44, respectively). Considering the relatively large effect size on self-reported measures in Study 1, such a sample would provide nearly certainty to detect a true effect on the self-reported measures. However, the effect-size found in Study 2 on the Stroop task is nearly silent concerning the estimated effect-size in the emotional sharing task. Consequently, power analysis assuming moderate effect size (f = .25) at alpha = .05 indicated that *n* of 132 would provide around 72% power (G*Power 3.1.9.2; Faul et al., 2009).

#### Hypotheses

The hypotheses (3.a, 3.b, 3.c, and 3.d) for self-reports measures in Study 3 are the same as the ones in Study 1.

Hypotheses regarding SSE reports are as follow:

Hyp. 3e: participants in the control condition will share more positive emotional reactions than participants in paternalistic and hostile conditions.

Hyp. 3f: participants in the paternalistic and hostile condition will share more negative emotional reactions than participants in the control condition.

Hyp. 3g: participants in the hostile condition will share significantly more negative than positive emotional reactions.

Hyp. 3h: participants in the paternalistic condition will share significantly more negative than positive emotional reactions.

Hyp. 3h_bis_: participants in the paternalistic condition will share an equal percentage of negative and positive emotional reactions.

#### Procedure and measures

***Paternalism induction.*** Participants were asked to read a paternalist, hostile or neutral scenario, which were exactly the same as in Study 1. After reading one of the three scenarios, participants had to report how they felt, on a list of 15 positive (e.g., enthusiastic, happy) and 53 negative (e.g., angry, skeptical) emotions and feelings, using a 7-point Likert scale, from 1 (*not at all*) to 7 (*totally*). As in Study 1, we ran a factor analysis on all of the emotional items. Two factors were extracted, explaining 56% of the total variance. All the emotional items with loadings greater than .45 were kept and we created one positive (11 items) and one negative score (45 items). The emotional scores presented a good internal consistency, α = 94 for positive emotions and α = .98 for negative emotions.

Participants were then asked to imagine that their best friend had sent them an email asking how their first day at work was going. Since the process of socially sharing emotional experience is more likely to be engaged with a close one (parent, friend, partner, etc.), we asked them to take the time to respond to one of their best friend’s emails. In a document opened on the computer screen next to them, the following sentence was written: “Imagine that you’re entering your office, just after your meeting with the director and your new colleagues. You open your email box and it contains an email from your best friend, who is asking you how your first day on the job is going. Since you’re alone in your office, you decide to take 5 minutes to answer. In the document open on the computer, write your answer to your friend’s email.” Using EMOTAIX (Piolat & Bannour, 2009) as a support, we identified the emotional words used by the participants. Since EMOTAIX only identifies words and not expressions, it sometimes allocated the wrong valence to an expression (e.g., “not nice” was coded as positive, as the “not” was not taken into account), two of the authors analyzed the texts in order to complement EMOTAIX’s findings. We created two complementary variables: a positive social sharing index (percentage of socially shared positive emotions) and a negative social sharing index (percentage of socially shared negative emotions). Before being fully debriefed and thanked, participants completed a manipulation check, assessing the paternalistic, hostile and neutral tone of the text describing their meeting with the company’s director and colleagues. They were asked to say how paternalistic, hostile and neutral, separately, they found the meeting, the director and the colleagues, separately, to be, using nine separate 7-point Likert scale ranging from 1 (*not at all*) to 7 (*totally*). We created an index of paternalism (α = .92), hostility (α = .94) and neutrality (α = .85) by computing the means of the corresponding items.

### Results and Discussion

#### Manipulation check

The paternalistic condition was perceived as more paternalistic (*M* = 6.13, *SD* = .82) than the control (*M* = 2.98, *SD* = 1.23) and hostile conditions (*M* = 2.23, *SD* = 1.79), with all *t*s > 13.25, *p*s < .001, and Cohen’s *d*s > 2.80. The latter two conditions differed significantly from each other, *t* (85) = –2.29, *p* = .02; Cohen’s *d* = .49. Similarly, the hostile condition (*M* = 5.60, *SD* = 1.29) was perceived as more hostile than the paternalistic (*M* = 1.79, *SD* = 1.10) and control conditions (*M* = 1.73, *SD* = .83), with all *t*s > 14.61, *p*s < .001, and Cohen’s *d*s > 3.18. The latter two conditions did not differ significantly from each other, *t* (84) = .28, *p* = .78; *d* = .06. The control condition was perceived as more neutral (*M* = 5.23, *SD* = 1.04) than the paternalistic (*M* = 2.08, *SD* = 1.11) and hostile conditions (*M* = 2.28, *SD* = 1.31), with all *t*s > 11.68, *p*s < .001, and Cohen’s *d*s > 2.49. The latter two conditions did not differ significantly from each other, *t* (83) = –.76 *p* = .45; *d* = –.16.

#### Main results

We used a 3 (condition: Paternalist vs. Hostile vs. Control) × 2 (emotion valence: Positive vs. Negative) × 2 (measure of emotions: Self-Reported vs. Socially Shared) MANOVA, with valence and emotion measure as within-subject variables. The dependent variables were standardized, but for the sake of clarity, the results are displayed with the original metrics.

Not surprisingly, due to standardization, no main effect of emotion valence, *F* (1, 129) = .021, *p* = .88, η^2^_p_ = .00, or of type of emotional measure, *F* (1, 129) = .00, *p* = .98, η^2^_p_ = .00, was found. A significant interaction between condition and valence was found, *F* (2,129) = 57.16, *p* < .001, η^2^_p_ = .47, as was a significant interaction between condition and type of emotional measure, *F* (2,129) = 6.87, *p* = .001, η^2^_p_ = .04. The three-way interaction directly tested our hypothesis that there would be a difference between self-reports and social sharing of emotions. As expected, the three-way interaction between Condition × Valence × Emotion measure was significant, *F* (2, 129) = 7.65, *p* = .001, η^2^_p_ = .11. To interpret this interaction, we performed separate condition by valence ANOVAs on each type of emotional measure.

**Figure 2:**
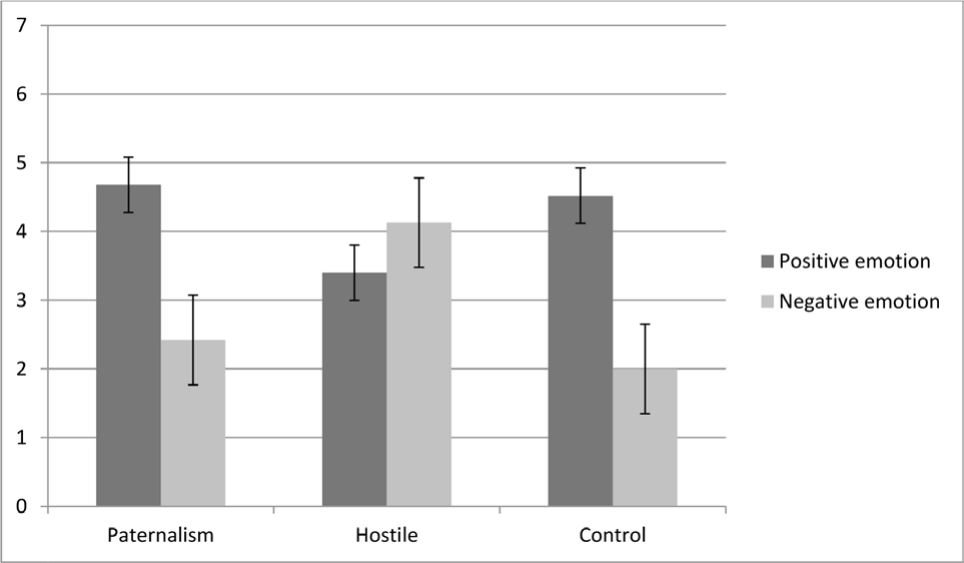
Positive and negative self-reported emotions by experimental condition (Study 3).

***Self-reported emotions.*** The analysis on positive and negative self-reported emotions revealed a significant main effect of valence, *F* (1,129) = 71.01, *p* <.001, η^2^_p_ = .35. Participants reported more positive (*M* = 4.21, *SD* = 1.24) than negative (*M* = 2.84, *SD* = 1.3) emotions, *t* (131) = 6.72, *p* < .001, Cohen’s *d* = 1.08, 95% CI [.97, 1.77]. As expected, the interaction between valence and condition was significant, *F* (2,129) = 41.96, *p* < .001, η^2^_p_ = .39 (see Figure 2). Post hoc analyses using the Bonferroni test indicated that participants in the paternalistic (*M* = 4.68, *SD* = 1.01) and control conditions (*M* = 4.52, *SD* = 1.08) reported significantly more positive emotions than those in the hostile condition (*M* = 3.40, *SD* = 1.22), with both *p*-values < .001, confirming Hyp. 3a. The mean score for reported positive emotions did not significantly differ between the paternalistic and control conditions, *p* = 1. Similarly, participants in the paternalistic (*M* = 2.42, *SD* = 1.04) and control (*M* = 2, *SD* = .85) conditions reported significantly fewer negative emotions than in the hostile condition (*M* = 4.13, *SD* = 1.14), with both *p*-values < .001, confirming Hyp. 3b. The mean score for reported negative emotions did not differ significantly between the paternalistic and control conditions, *p* = .16. In order to compare the level of positive and negative self-reported emotions within each condition, we performed paired t-tests. Participants in the paternalistic condition reported more positive (*M* = 4.67, *SD* = 1.01) than negative (*M* = 2.42, *SD* = 1.04) emotions, *t* (44) = 8.69, *p* < .001, *d* = 2.19, 95% CI [1.73, 2.77] (Hyp. 3c). As in the paternalistic condition, participants in the control condition reported more positive (*M* = 4.52, *SD* = 1.08) than negative emotions (*M* = 2, *SD* = .85), *t* (43) = 9.83, *p* < .001, *d* = 2.59, 95% CI [2, 3.04]. In the hostile condition, participants reported more negative (*M* = 4.13, *SD* = 1.14) than positive (*M* = 3.40, *SD* = 1.22) emotions, *t* (42) = –2.33, *p* = .025, *d* = .62, 95% CI [–1.36, –.01] (Hyp. 3d).

**Figure 3:**
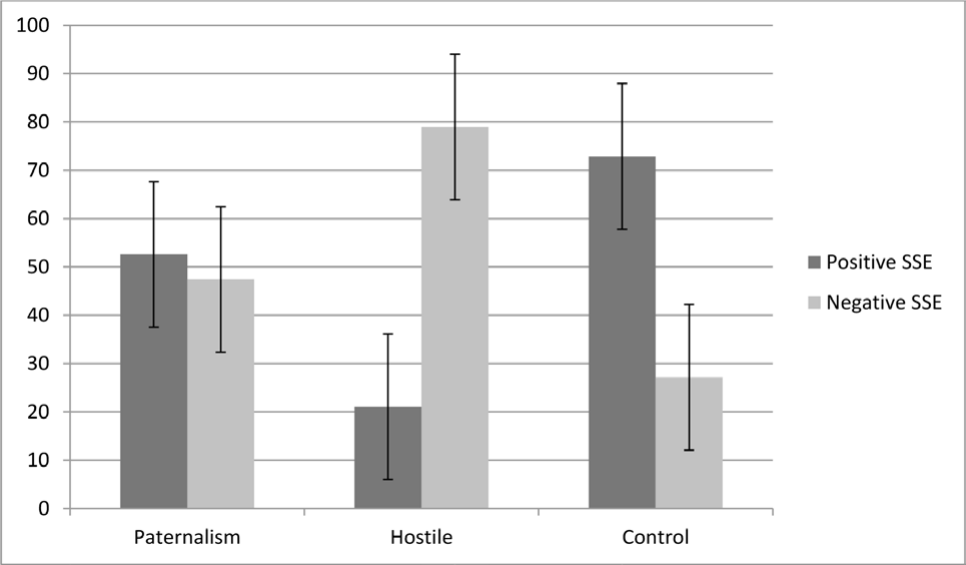
Percentages of positive and negative socially shared emotions by experimental condition (Study 3).

***Social sharing of emotions.*** The analysis of positive and negative socially shared emotions revealed no main effect of valence, *F* (1,129) = 3.11, *p* = .58, η^2^_p_ <.001. However, the interaction between condition and valence was significant, *F* (2,129) = 51.75, *p* < .001, η^2^_p_ = .44 (see Figure 3). Bonferroni post hoc analyses indicated that participants in the neutral condition socially shared more positive emotions (*M* = 72.87, *SD* = 22.82) than those in the hostile (*M* = 21.05, *SD* = 21.12) and paternalistic conditions (*M* = 52.59, *SD* = 27.26), both *p*s < .001, confirming Hyp. 3e. Also, Bonferroni post hoc analyses indicated that more negative emotions were socially shared in the hostile condition (*M* = 78.95, *SD* = 21.12) and paternalistic conditions (*M* = 47.41, *SD* = 27.26), compared to the control condition (*M* = 27.13, *SD* = 22.82), both *p*s < .001, confirming Hyp. 3f.

As with the self-reported emotions, we used paired t-tests to compare the percentage of socially shared positive and negative emotions within each condition. Participants in the hostile and control conditions presented the same pattern of results as observed in the analyses of self-reports, with more negative (*M* = 78.95, *SD* = 21.12) than positive (*M* = 21.05, *SD* = 21.12) emotions shared in the hostile condition, *t* (42) = –8.99, *p* < .001, *d* = 1.37, 95% CI [–70.90, –44.89] (Hyp. 3g), and more positive (*M* = 72.87, *SD* = 22.82) than negative (*M* = 27.13, *SD* = 22.82) emotions shared in the control condition, *t* (43) = 6.65, *p* < .001, *d* = 1, 95% CI [31.87, 59.62]. However, in line with Hyp. 3h_bis_, the percentage of positive socially shared emotions in the paternalistic condition (*M* = 52.59, *SD* = 27.26) did not differ significantly from the percentage of socially shared negative emotions (*M* = 47.41, *SD* = 27.26), *t* (44) = .64, *p* = .53, *d* = .09, 95% CI [–11.20, 21.56].

In summary, Study 3 did not provide evidence of a clear negative emotional state following paternalism. Based on Dardenne et al. (2007), which showed that paternalist sexism was perceived as unpleasant as hostile sexism, we were expecting that participants would socially share more negative emotions, compared to positive ones, in the paternalistic condition. Instead, our findings revealed that in the paternalistic condition, participants socially shared equal levels of positive and negative emotions. It seems that our participants felt more of an ambivalent emotional state rather than a clear negative one when confronted with paternalistic stereotypes.

## General Discussion and Conclusions

These studies examined why young victims of paternalistic stereotypes in the workplace do not systematically report them. We argued that, contrary to previous literature, the explanation does not relate to a failure to detect or identify paternalism’s negative aspect as such, but instead resides in the fact that the detection of reactions to paternalism is facilitated if more appropriate measures are used. The aim of this paper was to examine the relevance of more implicit measures to capture affective reactions of young professionals exposed to paternalistic stereotyping in a work-related context.

In a first study, we found that the use of explicit measures only apprehended the explicit positive message of paternalistic stereotypes. Participants reported more positive than negative emotions when confronted with paternalism, therefore missing out on the more subtle negative message of paternalism. The results of the second study showed that the use of an implicit emotional measure revealed that participants reacted to paternalism quite negatively. Indeed, in Study 2, the results revealed an attentional bias towards negative words in the paternalistic condition, compared to the control condition. Contrary to what could have been expected based on Dardenne et al. (2007)’s study, the results of our third study did not show that paternalistic stereotypes trigger only negative emotions. Instead, it seems that participants shared a more ambivalent affective state. This ambivalence is, nonetheless, not that surprising, given that paternalistic stereotyping conveys evaluations of incompetence using a positive tone. Because of the simultaneous presence of positive and negative views, paternalism is actually ambivalent by nature.

The main interest of the present studies was their focus on emotions. Previous research has demonstrated that paternalistic stereotyping has an impact on cognition, including working memory capacity (Beilock, Jellison, Rydell, McConnell, & Carr, 2006; Schmader & Johns, 2003), math performance (Gonzales, Blanton, & Williams, 2002; O’Brien & Crandall, 2003) and intrusive thoughts (Dumont et al., 2010). However, since the attitude has three components (i.e., cognition, emotion and behavior) that are only moderately correlated, knowing about the effect on cognition does not necessarily reveal much about the effects on emotion and on behavior. Our research starts to complement the literature, offering a more complete picture of the full impact of being “paternalised.”

In addition, our research complements the literature on targets of paternalistic stereotyping. Indeed, although most studies have focused on women, paternalism does not target women alone. Our research suggested that young and inexperienced workers can also live paternalism within the workplace and be left feeling ambivalent about it. Also, because they are perceived as warm but incompetent, elderly people can be targets of paternalistic stereotypes as well (Eckes, 2002; Fiske et al., 2002; Tuckett, 2006). Virtually any member of a social category that is stereotypically perceived as warm but incompetent can be the target of paternalism by another group or one of its members.

Finally, although it was not one of our initial aims, our studies fall in with the works of researchers taking an interest at the target’s perspective. In effect, research on the ambivalence created by paternalism has mainly studied the perpetrator’s perspective (Cuddy, et al., 2008; Dijker, 1987; Glick et al., 1996). Researchers were interested in explaining why people develop stereotypes of outgroup members (Fiske et al., 2002; Mackie & Smith, 2002). However, few have taken the target’s perspective. Some researchers showed that, even when impaired performance follows exposure to benevolent paternalism, its targets may have ambivalent attitudes. Targets may actually prescribe paternalism in some circumstances (Sarlet, Dumont, Delacollette, & Dardenne, 2012), and positive evaluation of the perpetrator has also been observed (Barreto & Ellemers, 2005). Our studies show that emotional and attitudinal ambivalence may not only be one of the causes of stereotyping attitudes but also one of the consequences.

Although our work begins to fill a gap in the literature, it does not explain the precise nature of the emotional reaction to paternalism. Because the factor analyses consistently revealed overall positive and negative factors, we can only speak in terms of overall negative and overall positive reactions. Since emotions do not all lead to the same action tendencies, investigating the exact components of the negative and positive reactions to paternalism could be very interesting. For instance, research has shown that anger encourages people to act against the source of negative stereotyping (Chaudoir & Quinn, 2010; Leonard, Moons, Mackie, & Smith, 2011), while fear acts in the opposite direction, leading people to flee from the source (Mackie, Devos, & Smith, 2000); meanwhile, contempt and disgust are related to avoidance (Brewer, 1999). Moreover, Mackie et al. (2000) showed in three studies that the responses to fear-related items were correlated with one another but were quite different from anger-related items, suggesting that knowing there is a general negative emotional reaction does not tell us much about exactly what is going on. Examining the impact of paternalism on specific positive and negative emotions might help us understand better and act better on these emotional reactions.

## Conclusions

One cannot blatantly stereotype someone without facing legal or social consequences. However, when the expression of the stereotype is less obvious, more subtle, fewer negative reactions are observed – sometimes none at all. We argue that this is not because the stereotype is so subtle that it is undetectable. The explanation seems to lie elsewhere. Using more appropriate measures to apprehend emotional reactions to subtly conveyed stereotypes appears to be a key. Whereas explicit measures capture explicit concepts, more subtle measures are needed to identify subtle concepts. However, the social consequences of reporting discrimination, albeit detected, can make people afraid to say anything, for fear of being perceived as drama queens making a big deal about nothing, especially if the discrimination is not obvious. Unfortunately, the fact that people do not dare to stand up against subtle discrimination does not mean that it cannot have harmful impacts on its targets. Consequently, even though subtle stereotyping is less obvious, it must be taken as seriously as blatant stereotyping.

## Footnotes

https://www.britsafe.org/speakupstaysafe.

The difference between the numbers of positive and negative emotion words is in line with the general preponderance of negative emotion words over positive ones in everyday language (Schrauf & Sanchez, 2004).
